# Potent O-antigen-deficient (rough) mutants of *Salmonella* Typhimurium secreting *Lawsonia intracellularis* antigens enhance immunogenicity and provide single-immunization protection against proliferative enteropathy and salmonellosis in a murine model

**DOI:** 10.1186/s13567-018-0552-8

**Published:** 2018-07-05

**Authors:** Suyeon Park, Gayeon Won, Jehyoung Kim, Hyeun Bum Kim, John Hwa Lee

**Affiliations:** 1 College of Veterinary Medicine, Chonbuk National University, Iksan Campus, Gobong-ro 79, Iksan, 54596 Republic of Korea; 2 Department of Animal Resources Science, Dankook University, Cheonan, 31116 South Korea

## Abstract

The obligate intracellular pathogen *Lawsonia intracellularis* (LI), the etiological agent of proliferative enteropathy (PE), poses a substantial economic loss in the swine industry worldwide. In this study, we genetically engineered an O-antigen-deficient (rough) *Salmonella* strain secreting four selected immunogenic LI antigens, namely OptA, OptB, LfliC, and Lhly. The genes encoding these antigens were individually inserted in the expression vector plasmid pJHL65, and the resultant plasmids were transformed into the *∆asd ∆lon ∆cpxR ∆rfaL Salmonella* Typhimurium (ST) strain JOL1800. The individual expression of the selected LI antigens in JOL1800 was validated by an immunoblotting assay. We observed significant (*P* < 0.05) induction of systemic IgG and mucosal IgA responses against each LI antigen or *Salmonella* outer membrane protein in mice immunized once orally with a mixture of four JOL1800-derived strains. Further, mRNA of IL-4 and IFN-γ were highly upregulated in splenic T cells re-stimulated in vitro with individual purified antigens. Subsequently, immunized mice showed significant protection against challenge with 10^6.9^ TCID_50_ LI or 2 × 10^9^ CFU of a virulent ST strain. At day 8 post-challenge, no mice in the immunized groups showed the presence of LI-specific genomic DNA (gDNA) in stool samples, while 50% of non-immunized mice were positive for LI-specific gDNA. Further, all the immunized mice survived the virulent ST challenge, compared to a 20% mortality rate observed in the control mice. Collectively, the constructed rough ST-based LI vaccine candidate efficiently elicited LI and ST-specific humoral and cell-mediated immunity and conferred proper dual protection against PE and salmonellosis.

## Introduction

Porcine proliferative enteritis (PE) caused by the obligate intracellular bacterium *Lawsonia intracellularis* (LI), which primarily occurs in grower or finisher pigs, poses a significant economic loss in swine-producing areas. The disease causes thickness of the intestinal mucosa due to hyperplasia of infected enterocytes. This leads to clinical signs such as lethargy, diarrhea, chronic enteritidis, cachexia, and sudden death caused by acute hemorrhagic enteritis. Considering the high prevalence of seropositive finisher pigs, estimated between 57 and 100% worldwide [1], effective intervention measures that can be applied to swine production systems need to be properly timed. An *L. intracellularis* modified-live vaccine (Enterisol^®^ Ileitis, Boehringer Ingelheim) is currently available and shows efficacy in reducing the severity of clinical symptoms in infected pigs. However, although currently available live attenuated LI vaccines have been shown to reduce clinical disease and to increase weight gain, however, these vaccines fail to induce complete protection against the LI challenge [2]. This live attenuated vaccine can be reactogenic, creating safety concerns. Furthermore, it is extremely difficult to differentiate between vaccinated and infected animals during diagnosis.

*Salmonella enterica* is a prevalent food-borne pathogen that is transmitted via contaminated meats. In particular, contaminated pork may account for approximately 25% of human salmonellosis cases [3]. Previous studies have revealed that LI infection is a risk factor for fecal shedding of *Salmonella* Typhimurium (ST) in affected pigs [4]. It has been reported that colonization and shedding of ST significantly increased in pigs experimentally co-infected with LI and ST [5]. This supports the hypothesis that both pathogens have indirect interactions that might be mediated by pre-disposing organisms, resulting in underlying changes in the composition of the intestinal microbiome [6]. Thus, a novel intervention strategy against both LI and ST is urgently needed to reduce the burden of enteric bacterial disease during pork production.

Current efforts to eradicate LI have been impeded by the difficulty associated with its in vitro cultivation. Very few antigenic determinants of LI, which can be exploited for the development of efficient vaccines, have been revealed. Recently, antibodies against the *Lawsonia* autotransporter A (LatA) protein were found in the sera of infected pigs [7]. This suggests that this protein is antigenic and is regularly encountered by the immune system in cases of natural infection. The LatA protein exists on the surface of the bacterium and is characterized by two highly immunogenic domains, optA and optB [8]. LI has an extracellular unipolar flagellum, which aids in the invasion of host cells and the broader infection of its natural hosts [9]. In LI, the genes involved in the biosynthesis of the flagellum are not yet fully identified. Our latest study showed that a putative flagellar-associated protein, LI0570, has flagellin-like traits and stimulates Toll-like receptor (TLR) 5-expressing immune cells. This leads to the production of proinflammatory cytokines and the subsequent activation of T cell-related adaptive immune responses [10]. Hemolytic and cytolytic activities are known to occur during natural LI infection in pigs [11]. Based on a sequence alignment, we recently characterized the antigenic features of the LI0004 protein, which we putatively defined as *Lawsonia* hemolysin A (LhlyA) [12]. This protein is highly similar to the RNA methyltransferase TlyA protein, a virulence factor in *Mycobacterium tuberculosis* that has dual activity as a hemolysin and an RNA methyltransferase [13]. Thus, we hypothesized that a vaccine based on LatA, flagellin (LfliC), and hemolysin (Lhly) could offer efficient protection against PE. Earlier studies have reported that live attenuated *Salmonella*-based vaccines carrying various heterologous bacterial or viral antigens elicit efficient humoral and cell-mediated immunity [14]. A *Salmonella*-based vaccination strategy is highly economical and allows for rapid deployment of vaccines. The O-antigen-deficient ST strain was evaluated for its efficacy as a carrier vector strain. A rough *Salmonella* variant [a mutant with a truncated lipopolysaccharide (LPS) O-antigen] has been tested as an attenuated vaccine candidate [15]. However, rough strains have rarely been employed as live vaccine delivery vector systems to date. One recent study reported that a rough *Salmonella* strain elicited an enhanced immune response via increased presentation in antigen-presenting cells, increased efficiency of uptake, and enhanced intracellular processing and degradation by dendritic cells (DCs) [16]. Improving the ST vector could circumvent problems with pre-existing anti-*Salmonella* antibodies in the vaccinated host. This would be achieved by the removal of the O-antigen of *Salmonella* LPS, a major bacterial surface immunoreactive component [17]. This strategy would also allow the differentiation of infected from vaccinated animals (DIVA), as antigenically and immunologically distinct antigens would be used for the vaccine. In this study, we constructed attenuated rough *S.* Typhimurium strains expressing and secreting OptA, OptB, LfliC, and Lhly. We evaluated the effectiveness of immunization with our constructs in vivo and in vitro using a murine model.

## Materials and methods

### Bacterial strains and plasmids used in this study

The bacterial strains and plasmids used in this study are listed in Table 1. The ST mutant strain JOL1800 (∆*asd* ∆*lon* ∆*cpxR* ∆*rfaL*) was grown in either Luria–Bertani (LB) broth or LB agar containing 50 μg/mL of diaminopimelic acid (DAP) (Sigma-Aldrich, St. Louis, MO, USA) at 37 °C. A balanced-lethal host-vector system was previously constructed by deletion of the auxotrophic gene aspartate–semialdehyde dehydrogenase (*asd*) in ST. This system provides antibiotic-free, stable maintenance of recombinant plasmids via *asd*^+^ complementation [18]. Live attenuated LI bacteria (Enterisol^®^ Ileitis) was purchased from Boehringer Ingelheim Vetmedica, Inc. St. Joseph, Missouri, USA, and resuspended in sterile phosphate buffered saline (PBS) to 10^6.9^TCID_50_ per 100 µL volume for the challenge studies.

**Table 1:** **Bacterial strains and plasmids used in this study**

Strain/plasmid	Description	Reference
*S*. Typhimurium		
JOL990	*S*. Typhimurium wild type, challenge strain	Lab stock
JOL1800	*Salmonella* Typhimurium ∆lon, ∆cpxR, ∆asd, ∆rfaL; bacterial delivery vector; rough strain	Lab stock
JOL1809	JOL1800 containing pJHL65 and expressing OptA	This study
JOL1810	JOL1800 containing pJHL80 and expressing OptB	This study
JOL1811	JOL1800 containing pJHL65 and expressing FliC	This study
JOL1812	JOL1800 containing pJHL65 and expressing Hly	This study
*E. coli*		
BL21(DE3)pLysS	F^−^, *omp*T, *hsd*S_B_ (r_B_^−^, m_B_^−^), *dcm, gal*, λ (DE3), pLysS, Cm^r^	Promega
JOL232	F^−^ λ^−^ ϕ80 ∆(*lacZYA*-*argF*) *endA1 recA1 hadR17 deoR thi*-*1 glnV44 gyrA96 relA1 ∆asdA4*	Lab stock
JOL1601	JOL232 containing pJHL80-OptA	This study
JOL1902	JOL232 containing pJHL65-OptB	This study
JOL1658	JOL232 containing pJHL65-FliC	This study
JOL1743	JOL232 containing pJHL65-Hly	This study
Plasmids		
pET28a(+)	IPTG-inducible expression vector; Kanamycin resistant	Novagen
pET32a	IPTG-inducible expression vector; Kanamycin resistant	Lab stock
pET-optA	pET32a derivative containing *optA*	This study
pET-optB	pET28a(+) derivative containing *optB*	This study
pET-fliC	pET28a(+) derivative containing *fliC*	This study
pET-hly	pET28a(+) derivative containing *hly*	This study
pJHL65	*asd*^+^ vector, pBR ori, β-lactamase signal sequence-based periplasmic secretion plasmid, 6xHis, high copy number	[24]
pJHL80	*asd*^+^ vector, p15A ori, β-lactamase signal sequence-based periplasmic secretion plasmid, 6xHis, high copy number	
pJHL-OptA	pJHL80 harboring *optA* of *L.* intracellularis	This study
pJHL-OptB	pJHL65 harboring *optB* of *L.* intracellularis	This study
pJHL-FliC	pJHL65 harboring *fliC* of *L.* intracellularis	This study
pJHL-Hly	pJHL65 harboring *hly* of *L.* intracellularis	This study
Primers		
rfaL DEL OT F	5′-GGATACGATAAACCGCAGTCG	This study
rfaL DEL OT R	5′- AACCGTGCGCTTGCTGATAAG	This study
rfaL DEL IN F	5′- ACAAGTTTAGGACTTCGCTGCC	This study
rfaL DEL IN R	5′-CAGAATGGTATTATGCGGACCG	This study

### Construction of a rough ST mutant delivering selected LI antigen proteins

The LPS biosynthesis gene ∆*rfaL* (encoding a molecular chaperone to facilitate interactions with the O-antigen ligase) was deleted from the JOL912 strain (∆*asd* ∆*lon* ∆*cpxR*) using a lambda red genome engineering technique, as previously described [19]. The knocked-out strain was designated JOL1800. Disruption of LPS synthesis in JOL1800 was validated by performing SDS-PAGE silver staining (data not provided). The gene sequence encoding LatA was divided into two highly immunogenic regions that were selected based on antigenicity and epitope accessibility using the BepiPred-2.0 web server [20, 21]. The OptA, OptB, LfliC, and Lhly gene sequences were obtained from the NCBI database and chemically synthesized (Bionee, Korea). The four selected LI antigen proteins were cloned in-frame downstream of the beta-lactamase signal sequence (*bla SS*) of the *asd*^+^ constitutive expression vector pJHL65 [22]. Subsequently, JOL1800 cells were electroporated with plasmids pJHL65-OptA, pJHL65-OptB, pJHL65-LFliC, or pJHL184-Lhly, resulting in the production of JOL1809, JOL1810, JOL1811, and JOL1812, respectively. The expression of each antigen protein in the recombinant strains was confirmed in the culture supernatants by immunoblotting assays [23] using polyclonal LI-specific hyperimmune sera raised against each protein in rabbits. The recombinant vaccine candidates (JOL1809, JOL1810, JOL1811, and JOL1812) were grown to mid-logarithmic phase in LB broth and were adjusted to yield approximately 1 × 10^8^ colony forming units (CFU) in sterile PBS (pH 7.4) for the purpose of inoculation. For each antigen protein purification, recombinant protein was expressed in pET28a and purified as previously described [23]. The LI specific protein was quantified using the Bradford assay and was confirmed by SDS-PAGE analyses.

### Animal experiments

All experimental work involving specific-pathogen-free BALB/c mice was performed under the approval of the Chonbuk National University Animal Ethics Committee (CBNU2015-00085) in accordance with the guidelines of the Korean Council on Animal Care and the Korean Animal Protection Law (2007, Article 13: Experiments with Animals). Five-week-old female BALB/c mice (*n* = 20) were randomly divided into two groups (*n *= 10). A single dose of the ghost vaccine candidate (total 1 × 10^8^ CFU of a mixture of the recombinant ST strains JOL1809, JOL1810, JOL1811, and JOL1812 at 2.5 × 10^7^ CFU each) was administered at week 0 into group B via the oral route. The mice in group A received only 100 μL of sterile PBS at week 0 and served as the control group. Whole blood and serum samples were collected from the retro-orbital sinus of each mouse. Vaginal and intestinal washes with PBS were also collected at weeks 0, 2, 4, 6, and 8 post-immunization (pi). The samples were stored at −80 °C to assess the LI-specific and *Salmonella* OmpA-specific serum IgG, intestinal IgA, and vaginal IgA humoral responses. For evaluating protection against ST infection, all mice were orally challenged with the virulent ST JOL990 strain (2 × 10^9^ CFU) at week 8 pi. Following the challenge, all mice were closely monitored for illness (such as diarrhea), abnormal behavior, survival, and weight loss until day 21 post-challenge. The spleens of all mice were collected aseptically and homogenized in 2 mL sterile PBS. A volume of 100 µL of each prepared homogenized spleen was spread onto brilliant green agar (BGA, Becton–Dickinson and Company) and incubated overnight at 37 °C. The CFUs are expressed as mean CFU ± SEM. For evaluating the T cell immune response elicited by the vaccine construct, an additional 10 mice (5 mice/group) were injected at week 0 using the same protocol described above. These mice were sacrificed for splenocytes at day 10 after immunization. The immune studies in BALB/c mice were performed for 6 weeks only. For challenge studies against LI infection, C57BL/6 mice were randomly divided into 6 groups (*n* = 30) vaccinated once orally with JOL1809, JOL1810, JOL1811, and JOL1812 or the combination of the vaccine candidates (a mixture of four recombinant ST strains). The 2.5 × 10^7^ CFU of each bacterial strain was used for vaccination. After 2 weeks post-vaccination, all vaccinated mice were orally challenged with 10^6.9^TCID_50_ live attenuated LI bacteria and stool samples (*n* = 5) at day 0, 3, 6, 7 and 8 post-challenge were collected for the analysis of LI-specific genomic DNA (gDNA) by one step PCR assay following the protocol described previously [24].

### ELISA assays

Indirect enzyme-linked immunosorbent assays (ELISA) were used to measure IgG and secretory IgA (sIgA) responses against OptA, OptB, LfliC, and Lhly in the sera (IgG) and in the vaginal and intestinal wash samples (sIgA), according to a previously described method [23]. We also assessed the *Salmonella*-specific IgG and sIgA responses against the *S*. Typhimurium outer membrane protein (OMP) in the immunized mice. Recombinant LI proteins (500 ng/well) and OmpA (250 ng/well) were used as the coating antigens for the determination of the LI-specific and *Salmonella*-specific antibody responses, respectively.

### FACS analysis

Following vaccination, the magnitude of the differentiation of splenic T cells was assessed using fluorescence-activated cell sorting (FACS), as previously described. Briefly, the mice were euthanized, and spleens were aseptically isolated at day 10 post-immunization. The prepared splenocytes (1 × 10^6^ cells/mL) were incubated with a mixture of fluorescent-labeled antibodies, such as anti-mouse CD3a-PE, anti-mouse CD4-perCP-vio700, and anti-mouse CD8a-FITC (Miltenyi Biotec, Bergisch Gladbach, Germany) for 15 min at 4 °C in the dark. The samples were washed twice with FACS buffer (Miltenyi Biotec) and resuspended in 200 μL of FACS buffer. The cells were then examined with the MACSQuant^®^ Analyzer (Miltenyi Biotec). The results were further analyzed using FlowJo Software (Tree Star Inc., CA, USA).

### Cytokine measurements

Following immunization, gene expression levels of the immunomodulatory cytokines interleukin-4 (IL-4), interferon-γ (INF-γ), and IL-17 were evaluated by reverse transcription real-time PCR (RT-PCR). Splenocytes (1 × 10^6^ cells/mL) isolated from the immunized mice were stimulated with 200 ng LI-specific antigens for 24 h, and then total RNA was isolated using the GeneAll^®^ Hybrid-R™ kit (GeneAll Biotechnology, Seoul, Korea), as per the manufacturer’s instructions. The cDNA was prepared from equal quantities of RNA (1 µg) using the ReverTra Ace^®^ qPCR RT kit (FSQ-101, TOYOBO, Japan), as previously described [25]. Samples were stored at −20 °C until use. RT-PCR for gene expression studies was performed using ABI Power SYBR Green PCR Master Mix (#4367659, Applied Biosystems, USA), as described previously [25]. All the primer pairs used in these analyses are described in a previous study [26]. The expression of mRNA was relatively quantified based on the expression of β-actin, using the 2^−∆∆CT^ method [27].

### Statistical analysis

Differences between the immunized and non-immunized groups were analyzed using t-tests (two-tailed) for two groups and one way ANOVA with post hoc Tukey’s multiple comparison test for more than two groups, and were expressed as mean ± standard error of the mean (SEM). *P*-values less than 0.05 were considered significant.

## Results

### Expression of the LI-specific antigens OptA, OptB, LFliC, and LHly in a rough *Salmonella* vector system

**Figure 1:**
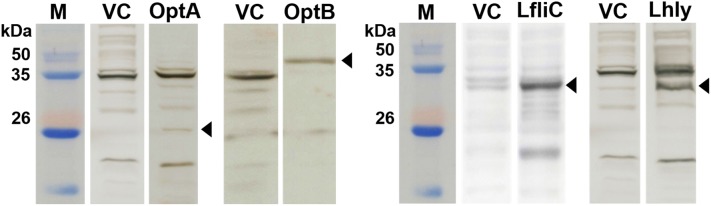
**Immunoblot analysis of optA, optB, LfliC and Lhly antigens expressed in JOL1800 derived strains, JOL1809, JOL1810, JOL1811 and JOL1812, respectively.** The respective vaccine strains were grown in LB broth to mid-log phase and then the culture supernatants were collected and subjected to Western blot analysis using protein-specific hyperimmune sera raised in rabbits. The expression plasmid, pJHL65 transformed into JOL912 was used as a vector control. The predicted molecular mass of the expressed recombinant proteins were ~17 kDa for OptA, ~41.1 kDa for OptB, ~38.6 kDa for FliC and ~30 kDa for Hly. Lane M, size marker; lane C, vector control; lane OptA, a pellet of JOL1809; lane OptB, a pellet of JOL1810; lane FliC, a pellet of JOL1811; lane Hly, a pellet of JOL1812.

Conserved domains of the LatA (LI0649) protein in *Lawsonia intracellularis* (PHE/MN1-00) were predicted using the BepiPred 2.0 program to assess potential antigenic features of the protein. To prevent overload of the secretion system during gene expression, two immunogenic regions in LatA, consisting of amino acid residues 101–200 and 534–851, were chosen based on epitope characteristics such as structural domains, hydrophilicity residues, antigenicity, and surface possibility and were designated *optA* and *optB*, respectively. The full-length versions of LfliC and Lhly were selected for expression in the *Salmonella* system. The gene sequences of these LI antigenic proteins were codon-optimized for efficient gene expression in the *Salmonella* system and then chemically synthesized. The insertion of *optA*, *optB*, *LfliC*, and *Lhly* into the pJHL65 vector was confirmed by digestion of the positive clones with *EcoR1* and *HindIII* to release fragments of approximately 300, 957, 885, and 756 bp, respectively. For construction of the rough *Salmonella* strain, the O-Ag ligase gene *rfaL* was deleted from the JOL912 genome to generate the ∆*lon*∆*cpxR*∆*asd*∆*rfaL* knockout mutant JOL1800. The deletion event was confirmed using outer and inner PCR primers that detect the flanking regions of the target gene. Upon replacement of wild-type *rfaL* with a *cat*^*R*^ gene cassette (~1.1 kb), the original amplicon size of 1.9 kb was reduced to 1.7 kb in the mutant-type. PCR primers designed to amplify the internal *rfaL* sequence yielded no amplification in the mutant strain (Table 1). The efficient secretion of each individual recombinant antigen in the rough *Salmonella* strain JOL1800 was validated by immunoblotting. The predicted molecular mass of the expressed recombinant proteins was 17 kDa for OptA, 41.1 kDa for OptB, 38.6 kDa for LfliC, and 30 kDa for Lhly (in the culture supernatants of JOL1809, JOL1810, JOL1811, and JOL1812, respectively, Figure 1).

### Humoral immune responses induced by vaccine candidates

**Figure 2:**
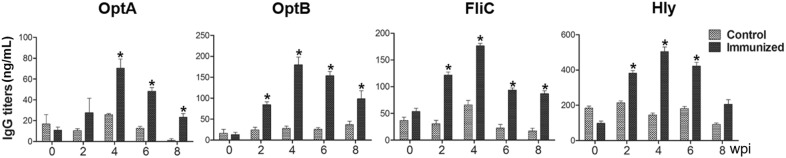
**Humoral immune responses specific to each purified LI antigen protein.** Titers of serum IgG were measured in the mice immunized with the mixture of JOL1800 derivatives. Control, a group inoculated with PBS; immunized, groups immunized with a combined formula of four JOL1800 derivatives. The error bars indicate the standard deviation (sd). wpi: week post-immunization; *P < 0.05 compared to titers of the control group.

**Figure 3:**
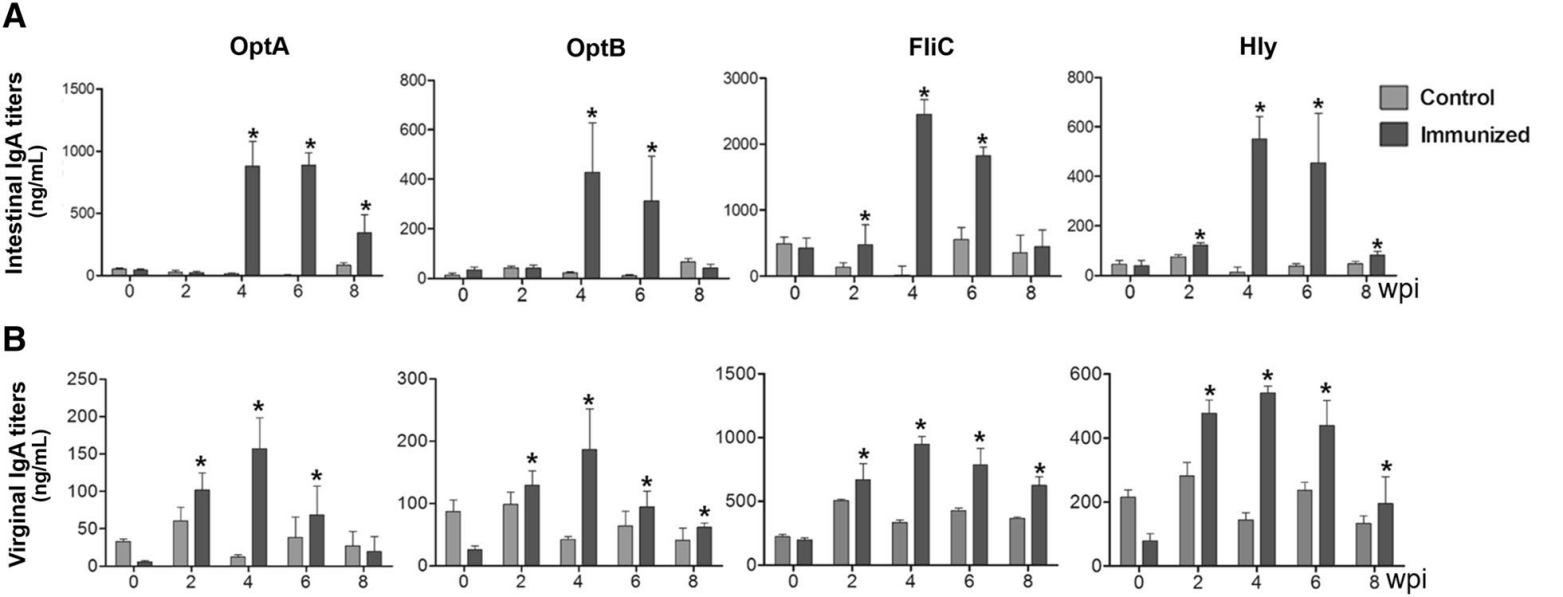
**Mucosal immune response.** Titers of intestinal secretory IgA (sIgA) (**A**) and virginal sIgA (**B**) obtained after vaccination with a co-mixture of all the four strains. The responses measured against each LI protein by an indirect ELISA using individual recombinant proteins as coating antigens. Each data points represent mean ± standard deviation (sd) of 10 mice/group. *P < 0.05. wpi: week post-immunization. ns: non-significant.

**Figure 4:**
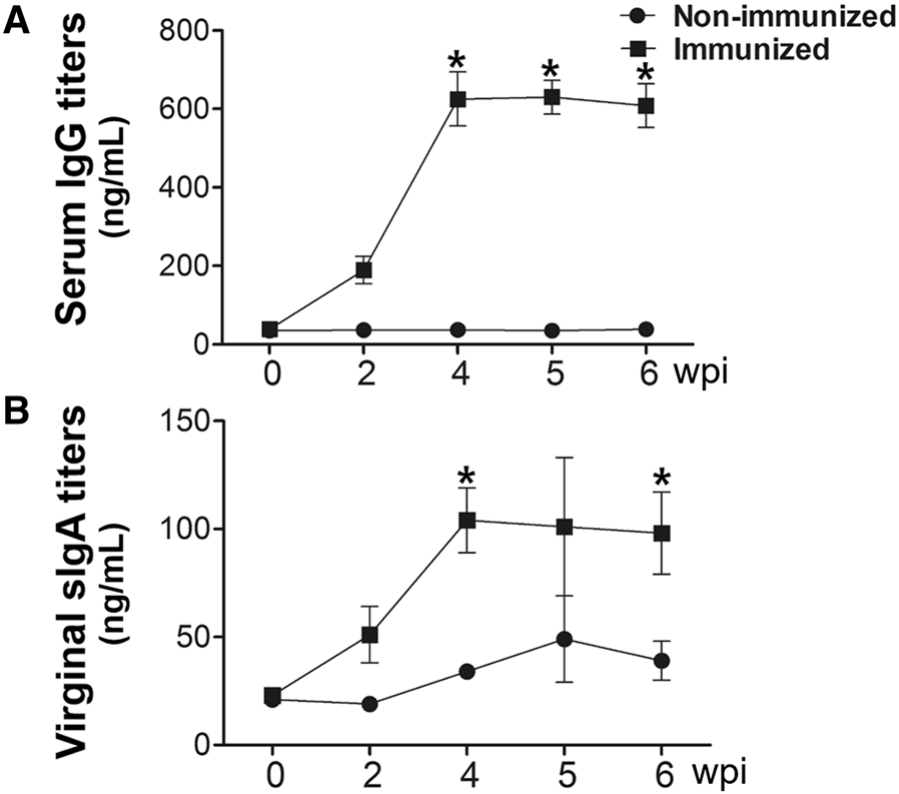
**Humoral and mucosal immune responses specific to the** ***Salmonella*** **OMP.** Titers of serum IgG titers (**a**) and virginal sIgA (**b**) after immunization with JOL1809, JOL1810, JOL1811 and JOL1812 in the mice via an oral route. Data are the means for all mice in each group (*n* = 10). PBS, antibodies elicited in the non-immunized mice; OptA, antibodies specific to OptA elicited by the immunization in the mice; OptB, antibodies specific to OptB elicited by the immunization in the mice; LFliC, antibodies specific to FliC elicited by the immunization in the mice; LHly, antibodies specific to Hly elicited by the immunization in the mice. Error bars indicate standard deviation (sd). wpi: week post-immunization; *P* < 0.05 (vs. PBS).

The ability of the rough ST mutant expressing LI antigens to induce systemic and mucosal antibody responses was evaluated in mice that had been orally immunized with a mixture of all four strains. All the immunized mice generated significantly increased levels of serum IgG against OptA, OptB, LFliC, and LHly at weeks 4 and 6 pi compared to the control group (*P* < 0.05) (Figure 2). The peak IgG titers were found at week 4 pi in all the groups and were maintained until week 6 or 8 pi. A significant elevation of IgA against OptA, OptB, LFliC, and LHly was also observed in both the intestinal and vaginal washes collected from the mice compared to those of the non-immunized group (Figure 3). In particular, increased sIgA secretion was detected in the intestinal washes compared to the vaginal washes, indicating that the candidate vaccine could provide a first line of defense against PE. ST-specific systemic IgG and mucosal IgA responses were also measured in the mice. Serum IgG titers specific to ST-OmpA markedly increased from week 4 pi through the end of the experiment (*P* < 0.05) (Figure 4A). Vaginal IgA titers also significantly increased in the immunized mice at weeks 4 and 6 pi (Figure 4B). The results implied that the rough ST mutant secreting LI antigens showed potential for generating antibodies capable of conferring dual protection against ST and LI.

### T cell-mediated immune responses

**Figure 5:**
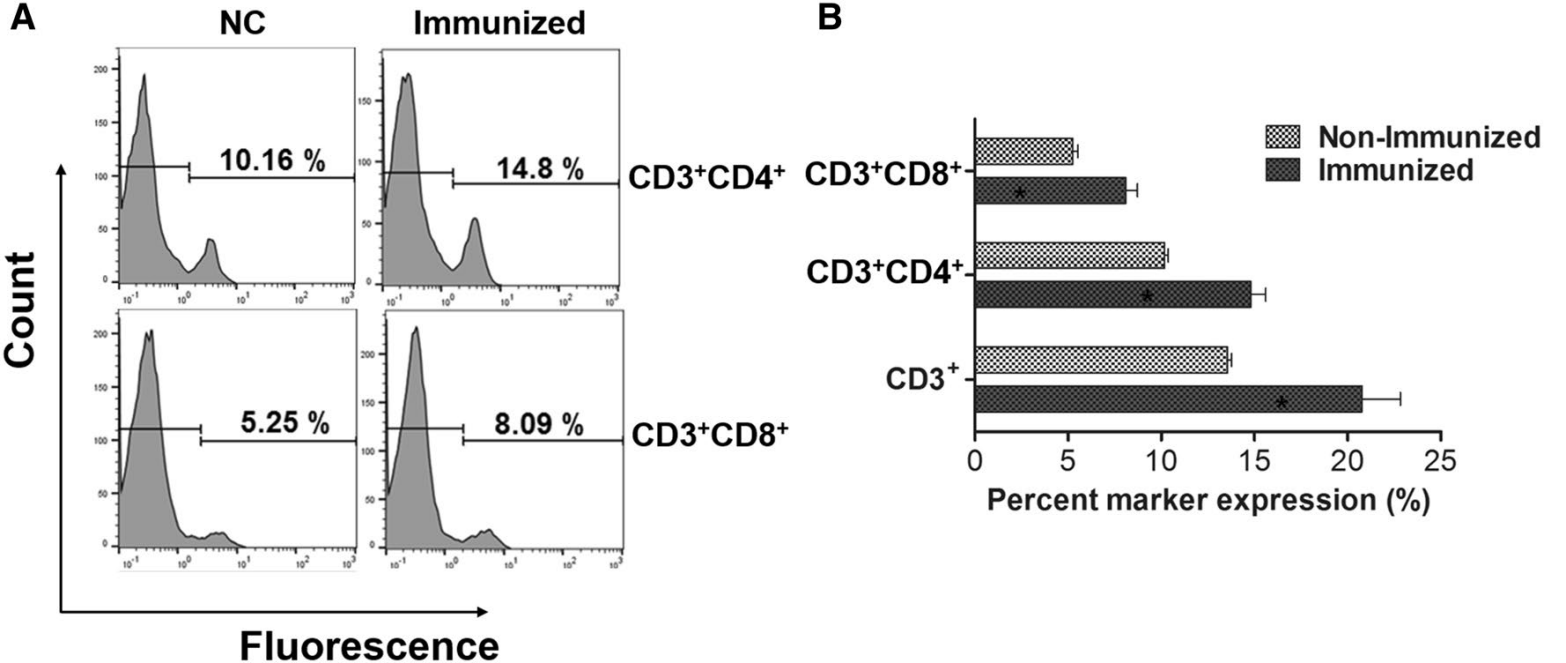
**FACS analysis of splenic T lymphocytes. A** Representative flow cytometry histogram plots for CD4^+^ and CD8^+^ splenic T cell populations. **B** Change in the T cell subpopulation in the control mice and immunized mice. Error bars indicate the sd. *P < 0.05 when the values were compared with those of non-immunized mice.

Altered proportions in CD3^+^, CD4^+^, and CD8^+^ T cell subpopulations were assessed using FACS analysis to evaluate the magnitude of differentiation among immunoregulatory T cells following vaccination. Markedly increased proportions of CD3^+^CD4^+^ and CD3^+^CD8^+^ T cells were observed in immunized mice compared to control mice (*P* < 0.05) (Figures 5A and B). Proportions of CD3^+^, CD3^+^CD4^+^, and CD3^+^CD8^+^ T cells increased by 3.3, 6.7, and 9.7%, respectively, in immunized mice compared to non-immunized mice (Figure 5B).

### Cellular immune responses and cytokine gene expression

**Figure 6:**
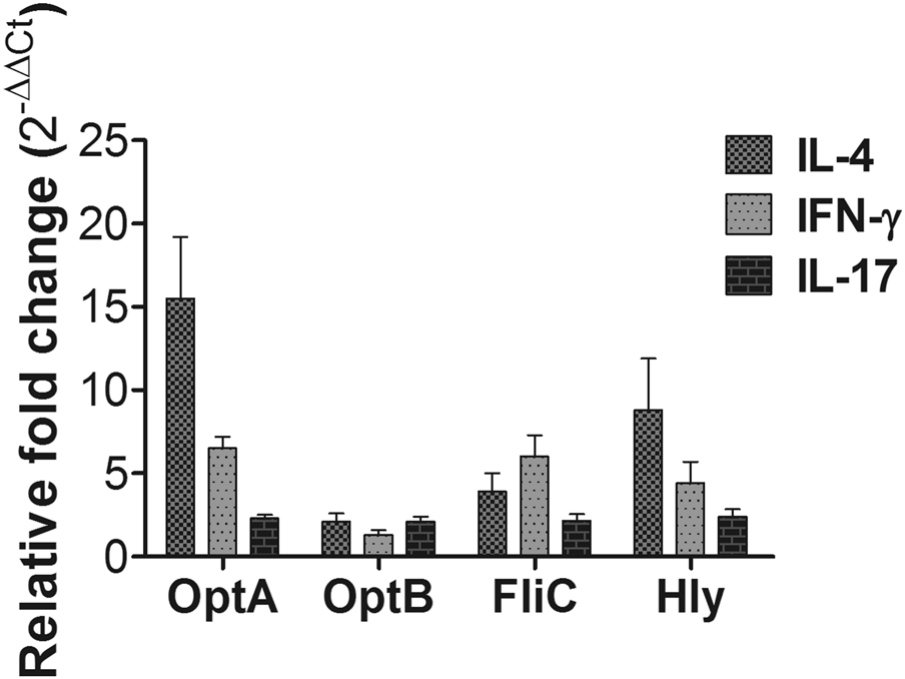
**Cytokine assay in splenocytes following in vitro re-stimulation.** The mRNA transcript levels of IL-4, IFN-γ and IL-17 were evaluated in the primed splenocytes pulsed in vitro with each antigen by performing RT PCR. The values of the relative fold change of each group were expressed as the mean ± SD. *P < 0.05 vs. the non-immunized control.

Immunomodulatory cytokines directly affecting the differentiation of effector T-helper (Th) cells were evaluated in splenic T cells isolated from mice using RT-PCR. To investigate the effect of rough ST-based LI vaccines on cytokine responses following immunization, mice were sacrifice at day 10 pi, and splenocytes were collected for analysis. Upregulation of pro-inflammatory cytokine mRNA was detected in splenocytes that were re-stimulated in vitro with OptA, OptB, LFliC, and LHly (Figure 6). The expression of both Th1-type (IFN-γ) and Th2-type (IL-4) cytokine mRNA was markedly augmented in the pulsed splenocytes compared to splenocytes isolated from control mice. These results suggest that vaccination with a rough ST-based L1 vaccine candidate can stimulate both Th1- and Th2-type immunity.

### Protection efficacy of the constructs against LI infection

**Figure 7:**
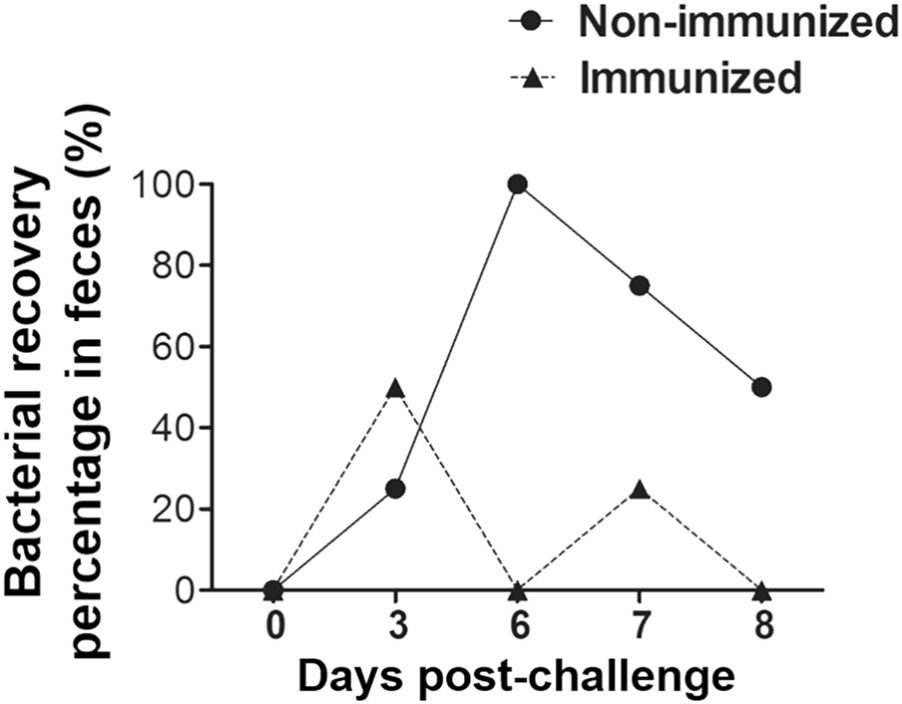
**Protection efficacies of four rough ST-based LI vaccines.** All the immunized and control mice groups were challenged with 10^6.9^TCID_50_ LI strains at week 8 post-immunization. Protection shown by a cocktail of all the four strains. The protection against LI was assessed based on the presence of gDNA in stool samples. Protection is presented as the number of animals showing gDNA in stool samples to the total number of animals in that group. Five animals were sacrificed at each time-point indicated.

To assess the protection offered by the *Salmonella*-based LI vaccine candidate, we challenged immunized mice orally with 10^6.9^ TCID_50_ live attenuated LI bacteria. Stool samples were analyzed by RT-PCR to evaluate whether genomic DNA (gDNA) of LI was present in the immunized mice following challenge. Our results demonstrated that the immunization with a cocktail of four strains significantly reduced LI gDNA in the mice by days 6 and 7 post-challenge. In the immunized groups, no mice showed gDNA in their stool samples while 100 and 50% of the control mice were positive for gDNA at days 6 and 8 post-challenge, respectively (Figure 7A). The results imply that vaccination with our construct has the potential to confer protection against PE.

### Protection efficacy of the constructs against ST infection

**Figure 8:**
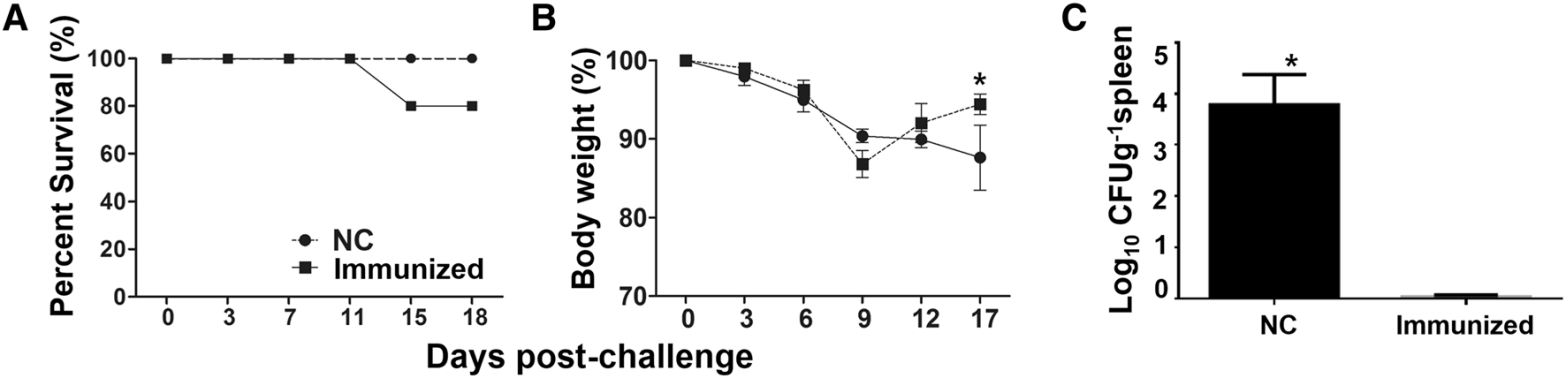
**Protection efficacies of** ***Salmonella*-based LI vaccines against a virulent** ***S.*** **Typhimurium.** Mice immunized with either *Salmonella*-based LI vaccine constructs or PBS were challenged at 6 weeks post-immunization with a virulent *Salmonella* strain and motility and bacterial recovery from spleen were recorded. **A** Motility in immunized and PBS control groups. **B** Percentage (%) in mouse body weight. **C** Bacterial load in survived mice post-challenge. Each data points represent mean ± standard deviation (sd) of 5 mice/group.

We next sought to determine whether immunization with a rough ST strain delivering LI antigens elicits protective immunity against wild-type ST infection. Immunized mice were administered an intraperitoneal inoculation (via the oral route) of a virulent ST strain, JOL990 (at the LD_50_), at week 8 pi. Mice immunized with our vaccine construct showed 100% survivability, while 20% mortality was observed in the PBS control group (Figure 8B). In both the immunized and non-immunized mice, weight loss was observed until day 9 post-challenge. While the weight of the immunized animals recovered by approximately 97% by day 17 post-challenge, that of the non-immunized animals declined until day 17 post-challenge (Figure 8A). At day 18 post-challenge, all mice were sacrificed, and the challenge strain was recovered from the spleens of the mice. Significantly less colonization was seen in the spleens from the immunized group (0.12 ± 0.03 log_10_ CFU/g) than in those from the control group (3.91 ± 0.74 log_10_ CFU/g) (Figure 8C). This indicates that rapid clearance occurred in the mice immunized with the vaccine construct.

## Discussion

In the present study, we report that an attenuated rough ST strain secreting select LI antigens (OptA, OptB, LFliC, and LHly) has the potential to provide immunogenicity and protection efficacy against both PE and salmonellosis. The causative agents of PE (LI) and salmonellosis (ST) are both invasive intracellular enteric bacteria. These pathogens have similar infection routes and resident niches, particularly in growing-finishing pigs. Co-infection with both pathogens results in severe symptoms, providing support for a synergistic interaction between them [6, 28]. Given the similar pathogenic mechanisms of these enteric pathogens, the construction of novel bivalent vaccines that can confer dual protection against PE and salmonellosis may contribute immensely to the swine industry by simultaneously preventing both diseases. In the initial stage of vaccine development, LI antigen proteins (OptA, OptB, LfliC, and Lhly) were chosen based on their antigenic profiles. These proteins are also putative virulence factors (e.g., outer membrane, flagellin, and toxin proteins) of LI [7, 10, 12]. For construction of the *Salmonella* vector system, the attenuated rough ST delivery strain JOL1800 was generated from a parental *lon*-*cpxR*-*asd* mutant ST strain, JOL912, via *rfaL* gene deletion. The *rfaL* gene is involved in the LPS core biosynthesis pathway, necessary for bacterial outer membrane biogenesis [29]. *Salmonella* serotyping is conventionally performed based on seroreactivity against O-Ag. In primed hosts, the major anti-*Salmonella* antibody is also generated against O-Ag. Use of the JOL1800 vector system for vaccine constructs in pigs should not interfere with LPS-based salmonellosis diagnostics and serotyping, as JOL1800 is DIVA-enabled [30]. Additionally, with the appropriate vaccine strain, preexisting immunity should not preclude the reuse of carriers or their use in areas where individuals have been previously exposed to *Salmonella* [31]. Some findings have suggested that ST vaccine vectors cannot be employed to deliver multiple doses of a vaccine antigen [32]. This is because the replication and spread of live vaccine strains can be curtailed by the presence of residual LPS antibodies or immunological memory from a prior immunization [32]. In our study, we anticipated that removal of any immunodominant LPS from the vector would minimize immune recognition and bacterial clearance in wild-type-primed hosts. Following vaccine construction, the efficient expression and secretion of each recombinant LI antigen protein in JOL1800 (using *bla SS*) were validated by Western blot analysis (Figure 1). Furthermore, increased titers of antibodies specific to each antigen in mice immunized with a mixture of JOL1809, JOL1810, JOL1811, and JOL1812 indicated that the strains sufficiently expressed each target antigen protein (Figures 2 and 3). Collectively, these results indicate that each target antigen was adequately secreted in the rough ST strains (i.e., the JOL1800-derived constructs), without significant conformational changes.

Active mucosal immunity plays a pivotal role in preventing invasion and intracellular proliferation of enteric pathogens [33, 34]. Mucosal sIgA helps antigen-presenting cells (APC) display epitopes to CD4^+^ T cells. This results in enhanced cell-mediated immunity, which is crucial for preventing invasion and intracellular proliferation of both LI and ST in the intestine [34–36]. Titers of secretory IgA (sIgA) specific to each LI antigen protein and to *Salmonella* OmpA were significantly elevated in the immunized group (Figures 3, 4). This indicates that the selected antigen proteins were efficiently presented to the organized lymphoid tissue of the mucosal immune system in the mice [37]. Considering that immunosuppression effects (such as decreases in T cell sub-populations) are observed in pigs heavily infected with LI [38, 39], vaccine candidates against LI should ideally induce robust cell-mediated immunity. Such immunity is required to defend against intracellular invasion and multiplication of enteric pathogens. A study by Cordes et al. showed infiltration of macrophages and CD8^+^ T cells in intestinal sections of pigs following LI infection [40], suggesting that cellular immune responses have a clear role in PE. A report by Smith et al. further showed that IFN-γ played an important role in limiting LI pathology in a mouse model [41]. In the present study, we observed a marked increase in the CD3^+^CD8^+^ T cell sub-population. These cells were likely significant effectors of antigen-specific, cell-mediated immunity (CMI) in the mice immunized with a mixture of four recombinant strains (Figure 5). We also observed an upregulation of IFN-γ mRNA in pulsed splenocytes following immunization. IFN-γ is a prerequisite autocrine growth factor for Th1-cells. A significantly higher IFN-γ response was observed in the group vaccinated with the cocktail of the rough ST strains (secreting the LI antigens) compared to the control group (Figure 6). Similarly, the number of mice showing LI infection was significantly lower (1/5) in the vaccinated group. Collins et al. also reported that rodents experimentally challenged with LI strain shed large numbers of the pathogen, which indicated the significant infection risk that rats pose to naïve pigs [42]. Thus, protective efficacy of the vaccination when given to the mice may show the potential to reduce PE prevalence. Importantly, vaccination with a *Salmonella*-based LI vaccine has the ability to offer concurrent protection against salmonellosis. In our study, meaningful protective efficacy against *S.* Typhimurium lethal challenge was observed in the immunized mice. This clearly implies that *Salmonella*-based LI vaccines can act as bivalent vaccines against salmonellosis and PE, thus making vaccination economical and cost-effective.

In conclusion, we demonstrated that a combination of all four strains induced efficient protection against both PE and salmonellosis in this study. This vaccine candidate induced significant increases in antigen-specific humoral, mucosal, and cellular immune responses in the immunized animals, providing synergistic, dual protection against PE and salmonellosis. Our study clearly showed that the vaccine candidate sufficiently conferred protective immunity against both PE and ST infection, likely by evoking a cell-mediated immune response. Further studies are needed in natural host pigs to evaluate the efficacy and potency of the rough ST-based LI vaccine. Studies to develop diagnostic tests for differentiating between infected and vaccinated animals (DIVA) are also warranted.
